# The ubiquitin ligase HERC4 suppresses MafA transcriptional activity triggered by GSK3β in myeloma by atypical K63-linked polyubiquitination

**DOI:** 10.1016/j.jbc.2023.104675

**Published:** 2023-04-05

**Authors:** Zubin Zhang, Mei Li, Peng Lin, Ying Ren, Yuanming He, Siyu Wang, Yujia Xu, Biyin Cao, Guanghui Wang, Michael F. Moran, Xinliang Mao

**Affiliations:** 1 Jiangsu Key Laboratory for Translational Research and Therapeutics of NeuroPsychoDiseases, Department of Pharmacology, College of Pharmaceutical Sciences, Soochow University, Suzhou, Jiangsu, China; 2 Institute of Pediatric Research, Children's Hospital of Soochow University, Suzhou, Jiangsu, China; 3 The Department of Molecular Genetics, The University of Toronto, Toronto, Ontario, Canada; 4 Guangdong Provincial Key Laboratory of Protein Modification and Degradation, State Key Laboratory of Respiratory Diseases, School of Basic Medical Sciences, Guangzhou Medical University, Guangzhou, Guangdong, China

**Keywords:** MafA, HERC4, K63-linked polyubiquitination, GSK3β, multiple myeloma

## Abstract

MafA and c-Maf are close members of the Maf transcription factor family and indicators of poor prognosis of multiple myeloma (MM). Our previous study finds that the ubiquitin ligase HERC4 induces c-Maf degradation but stabilizes MafA, and the mechanism is elusive. In the present study, we find that HERC4 interacts with MafA and mediates its K63-linked polyubiquitination at K33. Moreover, HERC4 inhibits MafA phosphorylation and its transcriptional activity triggered by glycogen synthase kinase 3β (GSK3β). The K33R MafA variant prevents HERC4 from inhibiting MafA phosphorylation and increases MafA transcriptional activity. Further analyses reveal that MafA can also activate the STAT3 signaling, but it is suppressed by HERC4. Lastly, we demonstrate that lithium chloride, a GSK3β inhibitor, can upregulate HERC4 and synergizes dexamethasone, a typical anti-MM drug, in inhibiting MM cell proliferation and xenograft growth in nude mice. These findings thus highlight a novel regulation of MafA oncogenic activity in MM and provide the rationale by targeting HERC4/GSK3β/MafA for the treatment of MM.

Multiple myeloma (MM) is an incurable plasma cell malignancy characterized by several chromosomal translocations associated with the heavy chain gene of immunoglobin (IgH) (1), of which t(6;14), t(8;14), and t(14;16) lead to the overexpression of MafB, MafA, and c-Maf, three close members of the Maf transcription factor family (2). As the basic leucine zipper transcription factors like c-fos and c-jun, Maf proteins promote transcription of their target genes including cyclin D2, CCR1, integrin beta 7 (ITGβ7), and ARK5 through their binding to the palindromic sequences called Maf recognition elements (3, 4). c-Maf transgenic mice can develop MM-like symptoms including hyperglobulinemia and associated kidney damage at 60 to 80 weeks old (3). MafA is also able to transform chicken embryo fibroblasts in a cell context-dependent manner (5). Dysregulation of the Maf proteins are recognized as one of the most important factors of poor prognosis in patients with MM (6).

The function and abundance of Maf proteins are controlled by multiple posttranscriptional modifications, such as phosphorylation (7), SUMOylation (8), and ubiquitination (4). The glycogen synthase kinase 3β (GSK3β) activates MafA and c-Maf *via* phosphorylation (9, 10). Ubiquitination not only modulates protein stability but also regulates protein function and subcellular localization. The ubiquitination process is regulated by three classes of sequential enzymes, including ubiquitin-activating enzymes (E1), ubiquitin-conjugating enzymes (E2), and ubiquitin ligases (E3). Our previous study identifies that the ubiquitin ligase HERC4 mediates c-Maf for K48-linked polyubiquitination and subsequent degradation (11). In contrast, HERC4 increases MafA stability (11). Given c-Maf and MafA share a high similarity in amino acid composition and belong to the same Maf protein family, we wonder the underlying mechanism in which HERC4 stabilizes MafA protein.

In the present study, we found that HERC4 increases the stability of MafA protein by inducing its K63-linked polyubiquitination at its Lys33 (K33). We further found that HERC4 interacts with GSK3β therefore inhibiting MafA phosphorylation and transcriptional activity. Inhibition of GSK3β by lithium chloride (LiCl) upregulates HERC4 expression therefore enhancing dexamethasone with antimyeloma activity. Taken together, the present study highlights a novel modality against MM by targeting the HERC4/GSK3β/MafA axis.

## Results

### HERC4 stabilizes MafA protein and suppresses its transcriptional activity

**Figure 1:**
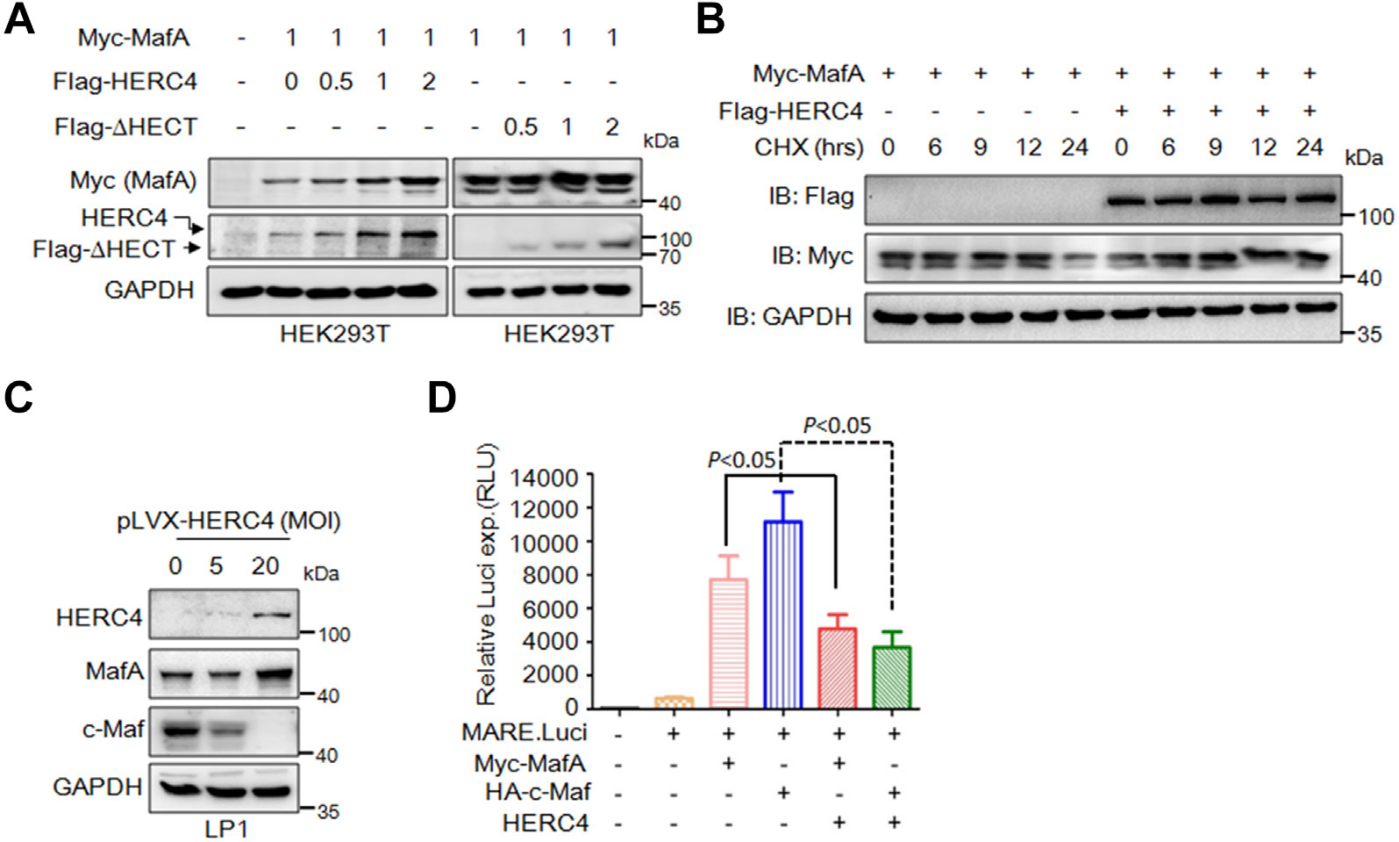
**HERC4 enhances thestability of MafA protein and suppresses its transcriptional activity.***A*, HEK293T cells were cotransfected with MafA and HERC4 or HERC4^ΔHECT^ for 48 h; cell lysates were then prepared for IB to measure MafA level. The pDsRed plasmid was used as a vector control. *B*, HEK293T cells were transfected with Myc-MafA alone or with HERC4. Forty-eight hours later, cells were treated with CHX (100 μg/ml) for 0, 6, 9, 12, and 24 h to inhibit *de novo* protein synthesis before being harvested for IB assays. *C*, LP1 cells were infected with HERC4 lentivirus for 72 h before being harvested for IB assays. *D*, the MARE.Luci plasmids were cotransfected with HERC4 and Myc-MafA or HA-c-Maf for 48 h followed by measurement of luciferase activity. CHX, cycloheximide; MARE, Maf recognition element.

HERC4 was found as a ubiquitin ligase to induce the ubiquitination and degradation of c-Maf, but it upregulates MafA at the protein level (11). To confirm this finding, we cotransfected Myc-MafA and Flag-HERC4 or Flag-HERC4^ΔHECT^ (a dead version of HERC4 not containing the HECT domain) plasmids into HEK293T cells; subsequent IB analysis revealed that HERC4, but not HERC4^ΔHECT^, markedly increased the protein level of MafA in a concentration-dependent manner (Fig. 1*A*). This finding was further confirmed in the cycloheximide (CHX) chase assay. As shown in Figure 1*B*, in the presence of CHX, HERC4 strikingly extended the half-life of MafA protein (Fig. 1*B*). Given HERC4 is gradually downregulated following the progress of myelomagenesis and it is lowly expressed in MM cells (11), we wondered whether overexpression of HERC4 could stabilize MafA in MM cells. As expected, overexpression of HERC4 markedly increased MafA but decreased c-Maf at the protein levels (Fig. 1*C*). Given MafA and c-Maf are oncogenic transcription factors in MM and HERC4 downregulated c-Maf but upregulated MafA proteins, we wondered its effect on MafA and c-Maf transcriptional activity. Consistent with the previous report and c-Maf degradation, HERC4 inhibited c-Maf transcriptional activity as assayed by using luciferase as the reporter (Fig. 1*D*). To our surprise, HERC4 also suppressed MafA transcriptional activity which was contradictory to MafA protein stabilization (Fig. 1*D*). Therefore, HERC4 upregulates MafA stability but inhibits its transcriptional activity.

### HERC4 interacts with MafA *via* the aa 376–728 fragment

**Figure 2:**
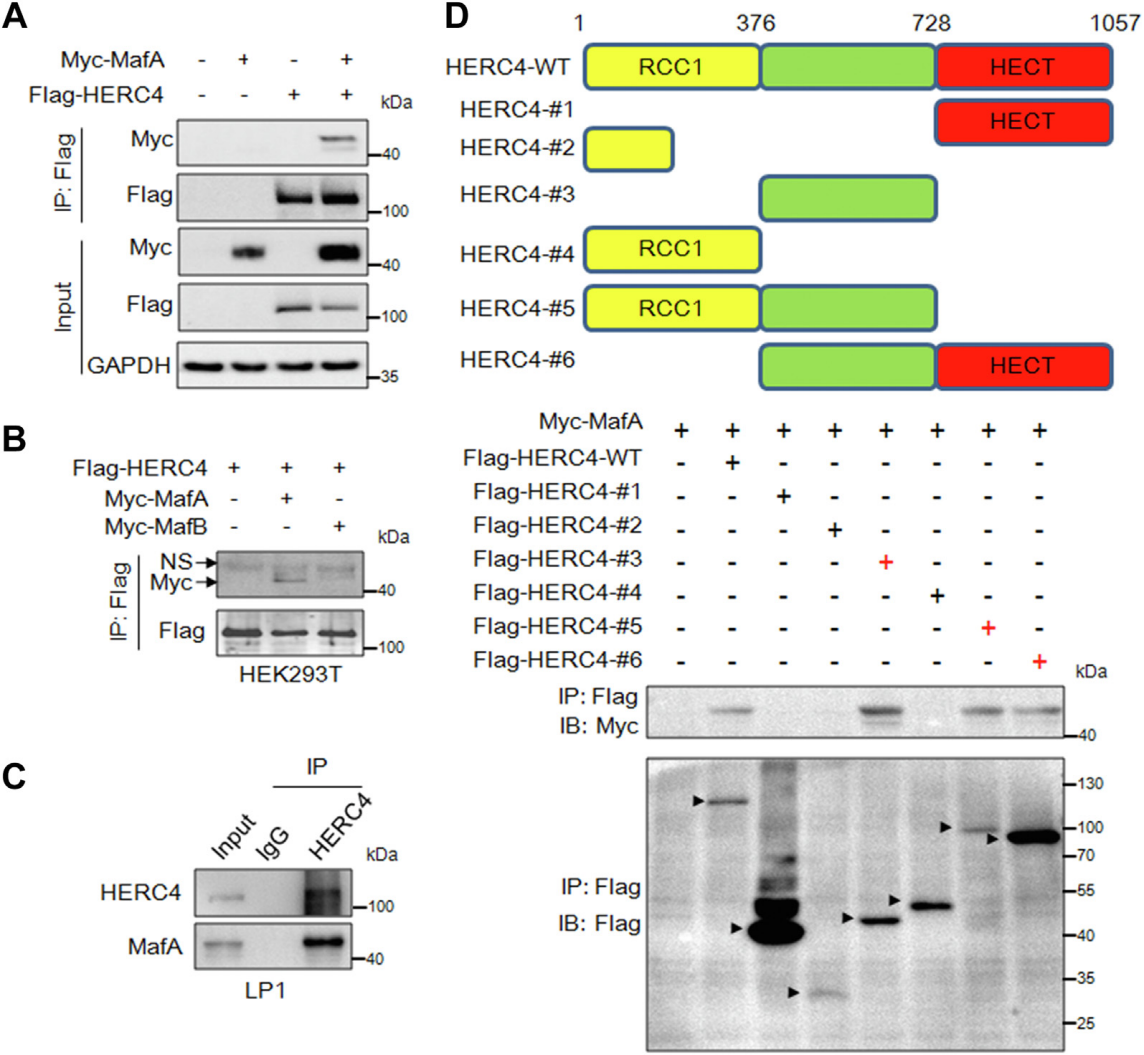
**HERC4 binds MafA at its aa 376–728 fragment.***A* and *B*, HEK293T cells were cotransfected with Flag-HERC4 and Myc-MafA (*A*) or Myc-MafB (*B*); 48 h later, cell lysates were prepared for IP with a specific antibody and IB assays with indicated antibodies. *C*, LP1 cell lysates were subjected to IP with anti-HERC4 and IB assays with anti-MafA. *D*, constructs of HERC4 truncates were cotransfected with Myc-MafA, respectively, for 48 h, followed by cell lysate preparation, IP and IB with specific antibodies as indicated. *Arrows* indicate individual truncated proteins. NS, nonspecific protein.

To elucidate how HERC4 stabilizes MafA but inhibits its transcriptional activity, we next wondered whether HERC4 as a ubiquitin ligase interacted with MafA. To this end, the MafA and HERC4 plasmids were cotransfected into HEK293T cells. The subsequent IP/IB assays revealed that MafA was identified by co-IP with ectopically overexpressed HERC4 (Fig. 2, *A* and *B*). Interestingly, HERC4 did not interact with MafB (Fig. 2*B*), which was consistent with that HERC4 did not alter MafB stability (11), suggesting HERC4 might prefer to interact with MafA and c-Maf. This interaction was also found in MM cells (Fig. 2*C*). To find the key domain by which HERC4 bound to MafA, a series of HERC4 truncates were generated and cotransfected with Myc-MafA into HEK293T. The subsequent IP/IB assay showed that only the truncates containing the aa 376–728 fragment interacted with MafA (Fig. 2*D*), suggesting that this aa 376–728 fragment was the binding region by which HERC4 interacted with MafA.

### HERC4 induces MafA for K63-linked polyubiquitination dependent on its HECT domain and C1025 residue

**Figure 3:**
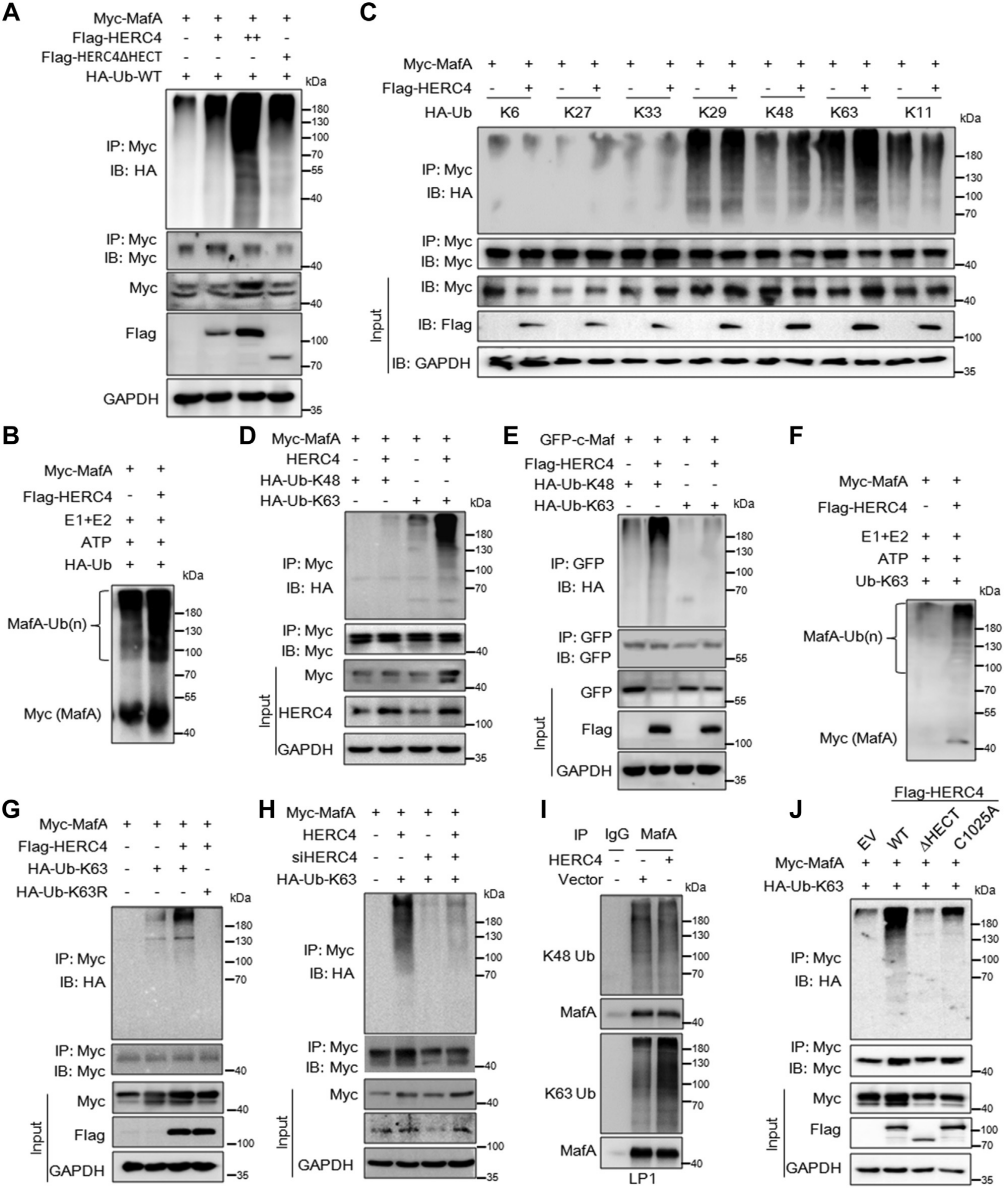
**HERC4 induces K63-linked polyubiquitination of MafA dependent on its HECT domain and Cys1025.***A*, HEK293T cells were transfected with Myc-MafA and HERC4 plasmids for 48 h followed by lysate preparation and IP/IB assays with specific antibodies as indicated. *B*, the purified Myc-MafA protein was incubated with E1, E2, ATP, and Ub-WT with or without purified Flag-HERC4 at 30 °C for 30 min. When the reaction was terminated, the reactions were subjected to IB against Myc-MafA. *C*, HEK293T cells were transfected with Myc-MafA and individual Ub mutants containing a single lysine residue (K) with or without a HERC4 plasmid. Forty-eight hours later, cell lysates were prepared for IP/IB assays as indicated. *D*, HEK293T cells were cotransfected with HERC4 and Myc-MafA, HA-Ub-K48, or HA-Ub-K63 for 48 h before being subjected to IP/IB assays with indicated antibodies. *E*, HEK293T cells were cotransfected with GFP-c-Maf and Flag-HERC4, HA-Ub-K48, or HA-Ub-K63 for 48 h before being subjected to IP/IB assays with indicated antibodies. *F*, the purified Myc-MafA protein was incubated with E1, E2, ATP, and K63-Ub with or without purified Flag-HERC4 at 30 °C for 30 min. When the reaction was terminated, the reactions were subjected to IB against Myc-MafA. *G*, HEK293T cells were transfected with HERC4, Myc-MafA, and HA-Ub-K63 or HA-Ub-K63R for 48 h before cell lysates were prepared for IP/IB as indicated. *H*, Myc-MafA, HERC4, and/or HERC4 siRNA were introduced into HEK293T cells; 48 h later, cell lysates were prepared for IP/IB assays as indicated. *I*, LP1 cells were infected with HERC4 lentivirus for 72 h, and the cell lysates were then subjected to IP/IB assays against specific proteins as indicated. *J*, HEK293T cells were transfected with Myc-MafA, HA-Ub-K63, and the WT or ΔHECT or C1025A HERC4. Forty-eight hours later, cell lysates were subjected to IP/IB assays as indicated.

Given HERC4 is a ubiquitin ligase and it interacts with MafA, we wondered whether it could direct MafA ubiquitination. To this end, the MafA ubiquitination levels were evaluated by the IP/IB ubiquitination assays *in vivo* and *in vitro*. The results showed in the presence of HERC4 and MafA was heavily polyubiquitinated (Fig. 3*A*). This ubiquitination was confirmed in the cell-free assay (Fig. 3*B*). Therefore, HERC4 mediates MafA polyubiquitination.

It is known that protein ubiquitination could occur at any lysine (K) residue of the ubiquitin molecule that leads to multiple forms of ubiquitination, including monoubiquitination and polyubiquitination including K48- and K63-linked, as well as K6-, K11-, K27-, K29-, and K33-linked forms (12), we wondered the specific ubiquitination form on MafA mediated by HERC4. To this end, plasmids containing MafA, HERC4, and individual ubiquitin mutants (HA-Ub-K6, HA-Ub-K11, -K27, -K29, -K33, -K48 and -K63) were co-transfected into HEK293T cells. Subsequent immunoprecipitation (IP)/immunoblotting (IB) assays showed that HERC4 mainly induced MafA K63-linked polyubiquitination, in addition to a slight K48-linked ubiquitination, but it failed to markedly increase K29- and K11-linked polyubiquitination (Fig. 3*C*). Therefore, we next focused on the K48- and K63-linked polyubiquitination on MafA by HERC4. To confirm this finding, plasmids containing MafA, HERC4, and K48-Ub or K63-Ub were cotransfected into HEK293T cells. The resultant IP/IB assay again showed that MafA displayed heavy ubiquitination with the K63-linked but slightly for K48-linked Ubiquitination (Fig. 3*D*). As a positive control, HERC4 mediated c-Maf for heavy K48-linked polyubiquitination but slightly K63-linked polyubiquitination (Fig. 3*E*), which was consistent with the previous report (11). Moreover, HERC4-mediated MafA for K63-linked polyubiquitination was confirmed in the *in vitro* ubiquitination system (Fig. 3*F*). Furthermore, when K63 was mutated (K63R-Ub), MafA failed to be ubiquitinated by HERC4 (Fig. 3*G*). Notably, when HERC4 was knocked down by its specific siRNA, MafA polyubiquitination was strikingly reduced (Fig. 3*H*). This finding was also recapitulated in MM cells in which lentiviral HERC4 mediated MafA for K63-linked but not K48-linked ubiquitination (Fig. 3*I*).

Because HERC4 belongs to the HECT family ligases, and the HECT domain is critical for its ubiquitin ligase activity (13), we wondered the role of the HECT domain in HERC4-mediated MafA ubiquitination. Therefore, we constructed two HERC4 plasmids, HERC4^ΔHECT^ that lacks the HECT domain and HERC4^C1025A^ in which the 1025th cysteine is located in the HECT domain and it is predicted as a key cysteine on its HECT domain (http://www.phosphosite.org). The parental HERC4 and mutants were cotransfected into HEK293T cells along with MafA and K63-Ub plasmids. The IP/IB assays found that, in contrast to the WT HERC4, both mutants lost its ubiquitin ligase activity to mediate MafA polyubiquitination (Fig. 3*J*). Taken together, all these findings demonstrate that HERC4 is a ubiquitin ligase for MafA K63-linked polyubiquitination, and its ligase activity depends on the HECT domain and C1025 residue.

### HERC4 suppresses MafA phosphorylation triggered by GSK3β *via* K63-linked polyubiquitination

**Figure 4:**
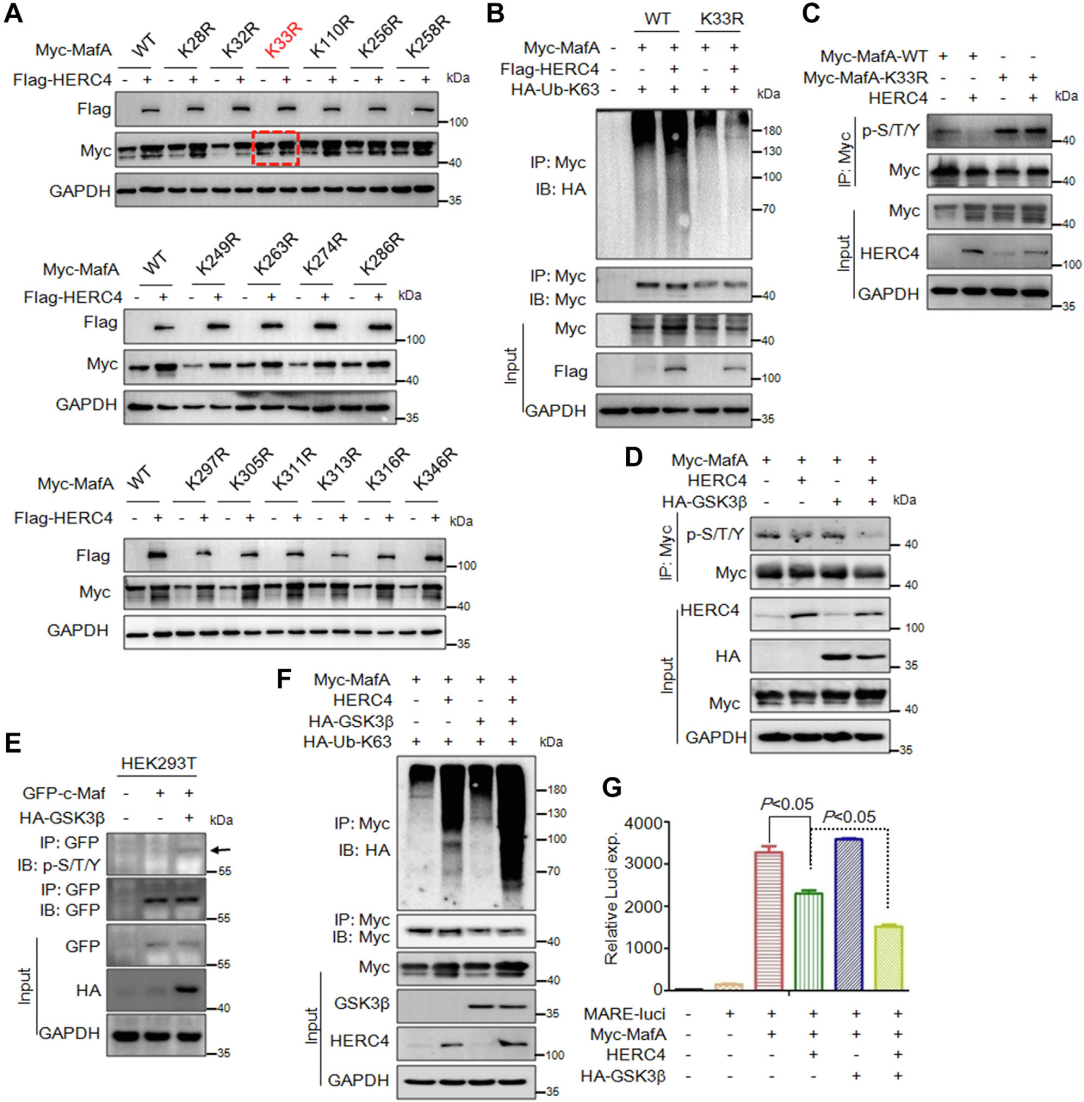
**HERC4 suppresses MafA phosphorylation triggered by GSK3β *via* K63-linked polyubiquitination.***A*, WT and mutant Myc-MafA plasmids (K-R) were transfected into HEK293T cells with or without a HERC4 plasmid. Forty-eight hours later, cell lysates were subjected to IB against Myc-MafA. *B*, HEK293T cells were transfected with Myc-MafA-WT, Myc-MafA-K33R, HA-Ub-K63 with or without a Flag-HERC4 plasmid. Forty-eight hours later, cells were followed by lysate preparation and IP/IB assays as indicated. *C*, HEK293T cells were transfected with HERC4 and WT or K33R MafA for 48 h, followed by IP with a Myc-specific antibody and IB with a p-S/T/Y antibody. *D*, HEK293T cells were transfected with HERC4, Myc-MafA, and/or HA-GSK3β. Forty-eight hours later, cells lysates were prepared for IP/IB as indicated. *E*, HEK293T cells were transfected with GFP-c-Maf and HA-GSK3β for 48 h, followed by IP with a GFP-specific antibody and IB with a p-S/T/Y antibody. *F*, HEK293T cells were transfected with HERC4, Myc-MafA, HA-GSK3β, and HA-Ub-K63. Forty-eight hours later, cells lysates were prepared for IP/IB as indicated. *G*, the MARE.Luci plasmids were cotransfected with Myc-MafA, HA-GSK3β, and HERC4 for 48 h followed by measurement of luciferase activity. GSK3β, glycogen synthase kinase 3β; MARE, Maf recognition element.

Ubiquitination occurs at the specific lysine (K) residue of a target protein; to identify which lysine residue(s) was a ubiquitin acceptor of MafA ubiquitination mediated by HERC4, we mutated MafA by K to arginine (R) substitution by using site-directed mutagenesis. These mutants were then cotransfected with HERC4 into HEK293T cells, followed by IB assays to monitor MafA protein stability. The results showed that HERC4 failed to upregulate K33R MafA but increased its WT and other K-R mutants (Fig. 4*A*), suggesting K33 might be the ubiquitin acceptor of MafA under the direction of HERC4. To confirm this finding, the K33R or WT MafA were cotransfected into HEK293T cells along with HERC4 and K63-Ub plasmids. The subsequent IP/IB assay showed that HERC4 mediated K63-linked polyubiquitination on the WT MafA but not its K33R variant (Fig. 4*B*), further suggesting that K33 is the ubiquitin acceptor of MafA mediated by HERC4.

It is reported that the transcriptional activity of MafA depends on its phosphorylation level (9, 14). To find out whether K63-linked polyubiquitination altered MafA phosphorylation and transcriptional activity, we next examined MafA phosphorylation in the presence of HERC4. We found that HERC4 overexpression decreased the phosphorylation level of WT but not K33R MafA (Fig. 4*C*), indicating HERC4 inhibited MafA phosphorylation in association with K63-linked polyubiquitination. Given GSK3β activates MafA by triggering its phosphorylation (9), we next evaluated the role of HERC4 on MafA phosphorylation in the presence of GSK3β. The IP/IB assay showed that GSK3β heavily phosphorylated MafA and it was inhibited by HERC4 (Fig. 4*D*), further suggesting that HERC4 indeed inhibited MafA phosphorylation. Moreover, GSK3β also increased the phosphorylation level (Fig. 4*E*), which was consistent with a previous report (7). To our surprise, GSK3β strikingly increased MafA for K63-linked polyubiquitination mediated by HERC4 (Fig. 4*F*), indicating GSK3β not only activated MafA phosphorylation but also promoted HERC4 ubiquitinating activity. Furthermore, the luciferase assays showed that GSK3β enhanced but HERC4 suppressed MafA transcriptional activity (Fig. 4*G*). In the presence of GSK3β, HERC4 displayed stronger inhibitory activity towards MafA (Fig. 4*G*). This finding is consistent with the inhibitory effect of HERC4 on MafA phosphorylation (Fig. 4*D*). Given MafA should be phosphorylated by GSK3β before its degradation (15), these findings suggested that when MafA was activated by GSK3β *via* phosphorylation, HERC4 was probably partially activated to induce MafA for K63-linked polyubiquitination upon its phosphorylation therefore limiting its transcriptional activity.

### HERC4 inhibits STAT3 transcriptional activity induced by MafA

**Figure 5:**
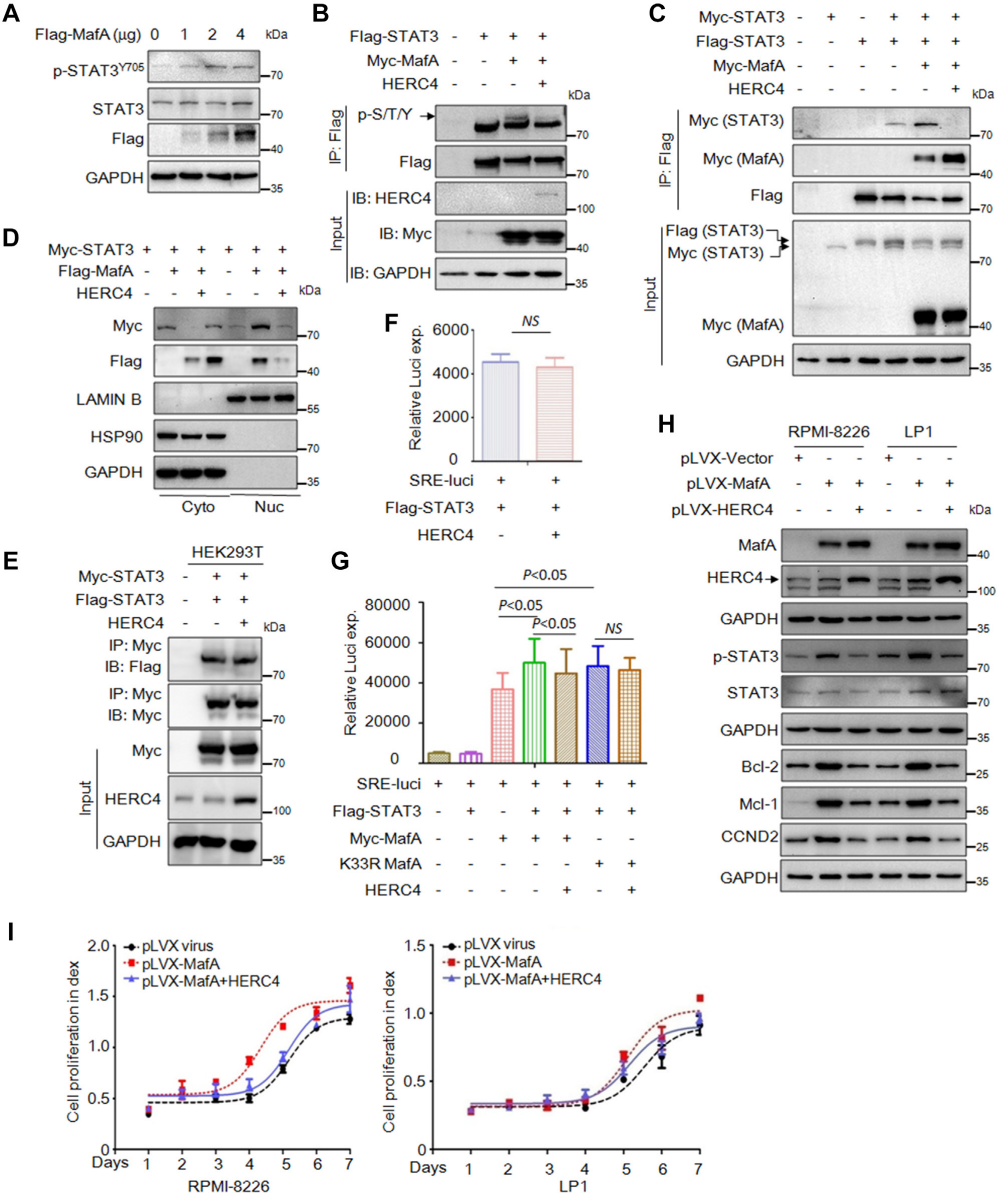
**HERC4 inhibits STAT3 transcriptional activity induced by MafA.***A*, HEK293T cells were transfected with a MafA plasmid for 48 h followed by cell lysate preparation and IB with indicated antibodies. *B*, HEK293T cells were transfected with STAT3, MafA, and HERC4 plasmids for 48 h, followed by cell lysate preparation and IP/IB with indicated antibodies. *C*, HEK293T cells were transfected with Myc- and/or Flag-STAT3, Myc-MafA, HERC4 for 48 h. Cell lysates were then prepared for IP/IB as indicated. *D*, HEK293T cells were transfected with Myc-STAT3, MafA, and HERC4 plasmids. Forty-eight hours later, cells were subjected to cell lysate preparation and nucleus-cytoplasm cellular fractionation, followed by IB analysis. *E*, HEK293T cells were transfected with Myc- and Flag-STAT3, HERC4 for 48 h. Cell lysates were then prepared for IP/IB as indicated. *F*, the p-STAT3.Luci plasmids were cotransfected with Flag-STAT3 and HERC4 for 48 h followed by the measurement of luciferase activity. *G*, the pSTAT3.Luci plasmids were cotransfected with Myc-MafA WT or K33R, Flag-STAT3, and HERC4 for 48 h followed by the measurement of luciferase activity. *H*, RPMI-8226 and LP1 cells were infected with MafA and HERC4 lentivirus for 96 h, followed by cell lysates preparation and IB assays against specific proteins as indicated. *I*, RPMI-8226 and LP-1 cells were infected with MafA and HERC4 virus; cell viability was measured by MTT assays.

Given the STAT3 pathway is very critical and highly activated in the progression of MM (16, 17) and there is a crosstalk between Maf protein and STAT3 in controlling human B cells (18), we wondered whether MafA could affect STAT3 activity. To this end, we transfected MafA into HEK293T cells with increasing concentrations; the subsequent assays showed that MafA markedly increased the phosphorylation level but not the total level of STAT3 (Fig. 5*A*). Moreover, the IP/IB results revealed that MafA induced STAT3 phosphorylation but it was inhibited by HERC4 (Fig. 5*B*), consistent with the inhibitory effects of HERC4 on MafA.

Because STAT3 activation requires its autodimerization before being translocated to the nuclei where it exerts its transcriptional activity, we wondered whether HERC4 and MafA affected STAT3 dimerization. To this end, we constructed a Myc- and a Flag-tagged STAT3 plasmid; these two plasmids were then cotransfected with MafA and HERC4 into HEK293T cells. The whole cell lysates were then subjected to IP/IB assays. It showed that MafA promoted STAT3 dimerization (Fig. 5*C*) and nuclear translocalization (Fig. 5*D*), but abolished by HERC4 (Fig. 5, *C* and *D*). Furthermore, given HEK293T did not express MafA, we next expressed HERC4 in HEK293T cells to examine the effects of HERC4 on STAT3; the IP/IB and luciferase results revealed that HERC4 alone failed to inhibit the dimerization and the transcriptional activity of STAT3 (Fig. 5, *E* and *F*). In contrast, MafA-upregulated STAT3 transcriptional activity was partly ablated by HERC4 in the assay of luciferase activity (Fig. 5*G*). These findings indicated that the effects of HERC4 on STAT3 require MafA. To solidify these findings in MM cells, lentiviral MafA and HERC4 were infected into RPMI-8226 and LP1, two typical MM cell lines expressing STAT3 (17), followed by the evaluation of STAT3 activity and the expression of its target genes by immunoblotting. The results showed that MafA markedly increased the phosphorylation of STAT3 and upregulated its target genes, such as Bcl-2, Mcl-1, and CCND2, but downregulated by HERC4 in both MM cell lines (Fig. 5*H*). Consistent with these findings, MafA promoted MM cell proliferation, but it was markedly suppressed by lentiviral HERC4 (Fig. 5*I*). Therefore, these results collectively suggested that MafA activates STAT3 phosphorylation, promotes its transcriptional activity and MM cell proliferation, but suppressed by HERC4.

### Suppression of MafA phosphorylation enhances dexamethasone efficacy in the treatment of MM *in vitro* and *in vivo*

**Figure 6:**
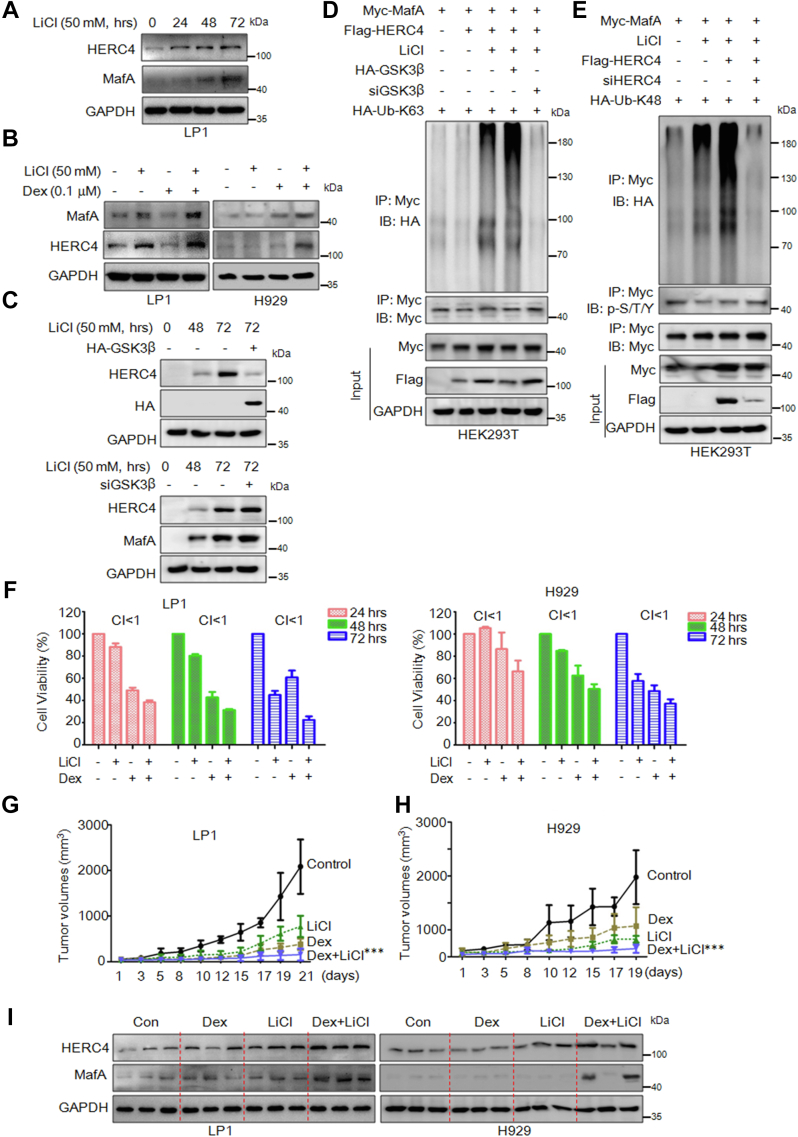
**Suppression of MafA phosphorylation enhances dexamethasone efficacy in the treatment of MM *in vitro* and *in vivo*.***A*, LP1 cells were treated with 50 mM lithium chloride (LiCl). Cell lysates were prepared at indicated periods for IB against indicated antibodies. *B*, NIH-H929 and LP1 cells were treated for 48 h with lithium chloride (LiCl), Dexamethasone (Dex) alone, or in combination. The cell lysates were subjected to IB assays as indicated. *C*, HEK293T and LP1 cells were transfected with HA-GSK3β and siGSK3β, respectively, followed by treatment of 50 mM LiCl for 48 and 72 h. *D*, HEK293T cells were transfected with Myc-MafA, Flag-HERC4, HA-Ub-K63, and HA-GSK3β plasmids or siGSK3β for 48 h, followed by treatment with LiCl. The resultant cell lysates were subjected to IP/IB with indicated antibodies. *E*, HEK293T cells were transfected with Myc-MafA, HA-Ub-K48, Flag-HERC4, or siHERC4 for 48 h, followed by LiCl treatment. Cell lysates were then prepared for IP/IB as indicated. *F*, NIH-H929 and LP1 cells were treated for 24, 48, and 72 h with indicated concentrations of LiCl, Dex alone, or in combination. Cell viability was determined using an MTT assay. Combination index (CI) less than 1 indicates synergy. *G* and *H*, NOD/SCID mice were inoculated subcutaneously with 2 × 10^7^ LP1 (*G*) or H929 (*H*) cells. When tumors were palpable, mice were divided randomly into four groups, of which each was injected intraperitoneally with LiCl, Dex alone, or in combination at a dose of 5 mg/kg body weight once a day. The same volume of vehicle was used as a control. Tumor sizes were monitored every other day. ∗∗∗*p* < 0.001, compared with the Dex group. *I*, tumor species were homogenized to prepare for cell lysates, followed by IB against indicated proteins. MM, multiple myeloma; GSK3β, glycogen synthase kinase 3β.

The aforementioned results demonstrated that HERC4 suppresses MafA phosphorylation and transcriptional activity driven by GSK3β. Previous studies have demonstrated that inhibition of GSK3β could induce MM cell death (7, 10); we wondered whether lithium chloride (LiCl), an inhibitor of GSK3β, could modulate HERC4 expression. To this end, LP1 cells were treated with increased LiCl, followed by immunoblotting assays. The results showed LiCl-upregulated HERC4 and MafA proteins in a concentration-dependent manner (Fig. 6*A*). Moreover, this effect was markedly enhanced by dexamethasone (Dex), a mainstay antimyeloma agent (Fig. 6*B*). In addition, when GSK3β was overexpressed or knockdown, the elevation of HERC4 was accordingly reduced or increased, respectively (Fig. 6*C*). To find out whether LiCl affected MafA ubiquitination mediated by HERC4, HEK293T cells were cotransfected with MafA, HERC4, K63-Ub, and GSK3β or siGSK3β, followed by LiCl treatment. The resultant IP/IB assays showed that the K63-linked polyubiquitination of MafA was strikingly increased by LiCl treatment, and interestingly, GSK3β overexpression enhanced MafA ubiquitination (Fig. 6*D*). Moreover, when GSK3β was knocked down, MafA ubiquitination was totally abolished (Fig. 6*D*). This assay thus suggested that LiCl- and HERC4-mediated MafA ubiquitination required GSK3β, this was consistent with the finding in Figure 4*F*. Moreover, we found that LiCl also markedly increased HERC4-mediated MafA K48-linked ubiquitination (Fig. 6*E*), suggesting GSK3β might be involved in both HERC4-mediated MafA ubiquitination at both K48- and K63-linked forms. We also found that the combined treatment of Dex and LiCl strikingly suppressed MM cell proliferation, in comparison with individual treatment (Fig. 6*F*). To evaluate the inhibitory effects of this combo *in vivo*, LP1 and NCI-H929 cells were used to establish human myeloma xenografts in immunodeficient nude mice, followed by treatment with LiCl, Dex alone, or in combination. The results showed that the combo administration almost suppressed the growth of xenografts in both models, more significant than the single treatment (Fig. 6, *G* and *H*). Notably, these alterations in tumor suppression were consistent with that the protein levels of both MafA and HERC4. The combo treatment strikingly upregulated HERC4 and MafA in the myeloma xenografts compared with the control or the single treatments (Fig. 6*I*).

## Discussion

The above studies find the E3 ligase HERC4 stabilizes MafA by inducing its K63-linked polyubiquitination. Moreover, this ubiquitination inhibits MafA transcriptional activity driven by GSK3β therefore suppressing MM cell proliferation and tumor growth. The HERC4/GSK3β/MafA axis could be a novel target for the treatment of a subset of MM patients with MafA overexpression.

MafA, c-Maf, and MafB belong to the Maf family transcription factors and their turnover is processed *via* the ubiquitin-proteasome pathway (4, 19, 20). Our recent study find that HERC4 is a ubiquitin ligase of c-Maf and promotes its degradation *via* K48-linked polyubiquitination, however, HERC4 stabilizes MafA and does not alter the protein level of MafB (11), indicating HERC4 may display a unique mechanism of action on MafA. The present study elucidates the underlying mechanism in which HERC4 binds to and stabilizes MafA by inducing its K63-linked polyubiquitination. Although K63-linked polyubiquitination is thought to modulate protein function and cellular distribution (21, 22), it also regulates protein stability. Recently, RNF6 is found to induce K63-linked polyubiquitination therefore stabilizing glucocorticoid receptor (23). Actually, in addition to K63-linked polyubiquitination, K27-linked ubiquitination also upregulates the protein stability (24). These findings further suggest that protein ubiquitination is much more complicated than what is known.

Beyond our expectation, HERC4 inhibits MafA transcriptional activity that contradicts to its increased stability. The underlying mechanism is also very interesting. Most studies found that there are crosstalks between protein phosphorylation and ubiquitination. In some proteins, their phosphorylation leads to ubiquitination and degradation, in contrast, in some cases, ubiquitination might modulate protein phosphorylation and biological activities. For example, ubiquitination promotes the phosphorylation of FANCI S/TQ cluster and activates the Fanconi Anemia I/D2 complex (25). In our study, HERC4-mediated K63-linked ubiquitination inhibits MafA transcriptional activity because K33, the ubiquitin acceptor for MafA mediated by HERC4, is mutated; MafA could not be ubiquitinated and its phosphorylation could not be inhibited by HERC4 (Fig. 4*C*). This finding also suggests there is a direct crosstalk between MafA ubiquitination and phosphorylation; K63-linked ubiquitination at K33 prevents MafA phosphorylation. HERC4 mediated MafA for K63-linked polyubiquitination therefore inhibiting the transcriptional activity of MafA. This could be the major mechanism in that HERC4 ubiquitinates and inhibits MafA. Furthermore, there are 16 lysine residues in the MafA protein; previous studies report that SUMOylation at K32 negatively regulate the function of MafA (8). Given K32 is proximal to K33, we wonder whether K33 ubiquitination enhances K32 SUMOylation therefore inhibiting MafA activity, but it should be further investigated.

Although HERC4 is oncogenically overexpressed in several cancers including lung cancer and breast cancer, it is downregulated during the progress of myelomagenesis from health bone marrow, MGUS, SMM to typical MM (11, 26). Moreover, restoration of HERC4 leads to c-Maf degradation and MM cell apoptosis (11). In the present study, we found that inhibition of GSK3β and induction of HERC4 might inhibit MafA and suppress myeloma cell proliferation and tumor growth. This is confirmed by the combined treatment of LiCl, the inhibitor of GSK3β (4), and Dex, a major anti-MM agent, which strikingly upregulate HERC4 and inhibit MafA therefore suppressing MM cell proliferation and myeloma xenograft growth in mice. This finding is of importance because the Maf proteins may contribute to resistance to some drugs that is used for MM therapy, such as proteasome inhibitors (10). Induction of HERC4 by chemical agents could downregulate c-Maf and inhibit MafA, which probably overcomes resistance contributed by the Maf proteins (10).

**Figure 7:**
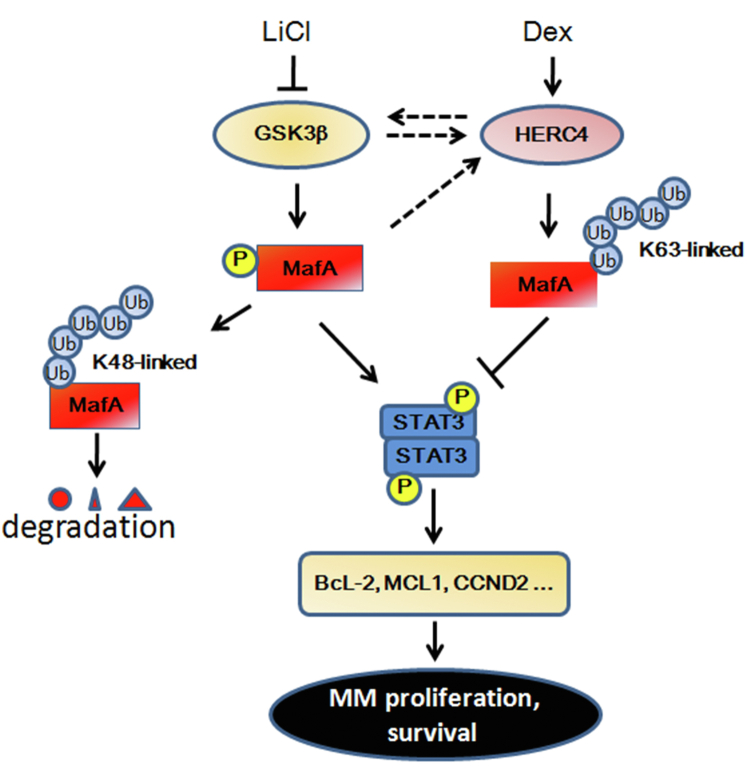
**Targeting the HERC4/GSK3β/MafA axis for the treatment of MM.** GSK3β is a kinase to activate MafA that further activates STAT3 and promotes its target gene expression. The phosphorylated MafA is further K48-linked ubiquitination and degraded. On the other hand, HERC4 directs MafA for K63-linked polyubiquitination at K33 that prevents it from phosphorylation by GSK3β. HERC4 therefore also inhibits STAT3 activation and transcriptional activity; inhibition of GSK3β will synergize induction of HERC4 for the treatment of MM. GSK3β, glycogen synthase kinase 3β; MM, multiple myeloma.

In summary, we demonstrate that HERC4 inhibits MafA by inducing its K63-linked polyubiquitination and inhibiting GSK3β-triggered MafA activity. HERC4 also inhibits the STAT3 pathway activated by MafA in MM cells. We further demonstrate that induction of HERC4 and inhibition of GSK3β could be a novel strategy for the treatment of MM with expression of MafA (Fig. 7). This study highlights a promising therapeutic modality of MM by targeting the HERC4/GSK3β/MafA axis. However, the detailed mechanism of HERC4 activity modulated by GSK3β should be further studied.

## Experimental procedures

### Cell culture

Human MM cells RPMI-8226, LP1, NCI-H929 were maintained in the lab and cultured in Iscove’s modified Dulbecco’s medium (HyClone); human HEK293T cells were cultured in Dulbecco’s high glucose modified Eagle’s medium (HyClone). All the media were supplemented with 1% penicillin/streptomycin solution (Beyotime Biotechnology). Cells were incubated at 37 °C in 5% CO_2_ atmosphere.

### Antibodies and plasmids

Rabbit anti-HERC4 and Rabbit anti-MafA antibodies were obtained from Bethyl Laboratories. Mouse anti-HA, anti-Myc, anti-Flag, and anti-GAPDH antibodies were obtained from Medical & Biological Laboratories Co Ltd. Anti-phosphoserine/threonine/tyrosine antibody was purchased from Abcam. Antibodies against GSK3β, c-Maf, and GFP were obtained from Santa Cruz Biotechnology. Antibodies against LAMIN B, HSP90, p-STAT3, STAT3, Bcl-2, Mcl-1, CCND2, K48-Ub, and K63-Ub were purchased from Cell Signaling Technologies. The HERC4 plasmid was subcloned into a pcDNA3.1 vector carrying a Flag or Myc tag (11). The HA-Ub, HA-Ub-K48, HA-Ub-K48R, HA-Ub-K63, and HA-Ub-K63R plasmids were constructed in-house (27). The human MafA gene was cloned from genomic DNA of HeLa cells as described previously (11).

### CHX chase assay

After transfected with plasmids for 24 h, HEK293T cells were treated with CHX (100 μg/ml, Sigma-Aldrich) for 0 to 24 h. Cell lysates were then prepared by using RIPA lysis buffer, followed SDS-PAGE and immunoblot analyses with specific antibodies as needed (11).

### siRNA transfection

HEK293T cells were transfected with HERC4 or GSK3β siRNA (GenePharma) using Lipofectamine RNAi^MAX^ (Thermo Fisher Scientific) following the manufacturer’s instruction.

### Luciferase reporter assay

The MAF and STAT3 recognition element-driven luciferase reporter were constructed, respectively, in pGL4 plasmids as described previously (11, 17). After the plasmids were transfected into HEK293T cells, cells were cultured for another 48 h and cell lysates were collected for the measurement of luciferase activity as described previously (11).

### MafA ubiquitination *in vitro*

Myc-MafA and Flag-HERC4 were separately expressed in HEK293T cells and were purified with specific antibodies against Myc and Flag, respectively, as described previously (11). After strict wash, MafA and HERC4 were eluted from the agarose beads before adding to the reaction mixture containing E1, E2, HA-Ub or Ub-K63, and ATP (Boston Biochem). The reaction was then terminated and subjected to immunoblot assay for MafA ubiquitination with specific antibody.

### Constructs of MafA with K to R mutation

Each construct of MafA with single lysine (K) to arginine (R) mutation was generated by using a PCR-based site-directed mutagenesis under direction of TransStart Fast pfu DNA polymerase (TransGen) as described previously (20) by mutating lysine codes (AAA or AAG) to arginine codes (AGA or AGG) where it was applicable. At the end of the PCR, the reactions were treated for 30 min by DpnI (Thermo Fisher Scientific), followed by transformation into DH5α competent cells (TIANGEN) and cultured in LB agar plates containing ampicillin (Beyotime).

### MafA lentivirus

MafA cDNA was generated by using the following primers: 5′-CCTCGAGCCACCATGGCCGCGGAGC-3′ (forward) and 5′-CGGATCCGCAGGAAGAAGTCGG-3′ (reverse), and the product was then inserted into pLVX-AcGFP lentiviral vector (Clontech) within the XhoI and BamHI sites. To generate lentiviral particles, HEK293T cells at 80% confluence were transfected with 10 μg of pLVX-AcGFP-HERC4, 3.5 μg of VSV-G envelope glycoprotein, 2.5 μg of packaging proteins (Rev), and 6.5 μg of packaging proteins (DR8.74) using Lipofectamine 2000 (Thermo Fisher Scientific). Cells were washed and refreshed with Dulbecco’s modified Eagle medium 12 h later. The lentiviral particles–enriched supernatant was harvested 48 h later, filtered, and stored at −80 °C. The titration of viral particles was determined by flow cytometry based on GFP expression. The pLVX-AcGFP lentiviral particles were used as a mock control in the infection assay.

### MM xenograft mice models

LP1 and NCI-H929 cells were suspended in 100 μl of PBS and inoculated subcutaneously into the flanks of NOD/SCID mice (n = 5 for each group, 5–6 weeks old) from Shanghai Slac Laboratory Animal Co Ltd. When tumors were palpable, mice were divided randomly into four groups. Mice were then injected intraperitoneally with dexamethasone (Sigma) at a dose of 5 mg/kg body weight and/or lithium chloride (LiCl) (Sigma) at a dose of 5 mg/kg body weight or vehicle of the same volume for 21 or 19 consecutive days. The tumor sizes were monitored every other day and calculated as described previously (11). At the end of the experiment, all mice were sacrificed and tumor tissues were removed, weighed, and lysed for immunoblotting assay. This study was approved by the Review Board of Animal Care and Use of Soochow University.

### Statistical analysis

Student’s *t* test was used to calculate *p* values for differences. Differences were considered significant at *p* < 0.05.

## Data availability

All data are contained within the manuscript.

## Conflict of interest

All authors declared no conflict of interest related to this study.
